# Detection and quantitation of copy number variation in the voltage-gated sodium channel gene of the mosquito *Culex quinquefasciatus*

**DOI:** 10.1038/s41598-017-06080-8

**Published:** 2017-07-19

**Authors:** Walter Fabricio Silva Martins, Krishanthi Subramaniam, Keith Steen, Henry Mawejje, Triantafillos Liloglou, Martin James Donnelly, Craig Stephen Wilding

**Affiliations:** 1 Department of Vector Biology, Liverpool School of Tropical Medicine, Liverpool, UK; 2 Departamento de Biologia, Universidade Estadual da Paraíba, Campina Grande, Brazil; 3 Infectious Diseases Research Collaboration, Kampala, Uganda; 4 Department of Molecular and Clinical Cancer Medicine, Roy Castle Lung Cancer Research, Liverpool, UK; 5 Malaria Programme, Wellcome Trust Sanger Institute, Cambridge, UK; 6 School of Natural Sciences and Psychology, Liverpool John Moores University, Liverpool, UK

## Abstract

Insecticide resistance is typically associated with alterations to the insecticidal target-site or with gene expression variation at loci involved in insecticide detoxification. In some species copy number variation (CNV) of target site loci (e.g. the *Ace-1* target site of carbamate insecticides) or detoxification genes has been implicated in the resistance phenotype. We show that field-collected Ugandan *Culex quinquefasciatus* display CNV for the voltage-gated sodium channel gene (*Vgsc*), target-site of pyrethroid and organochlorine insecticides. In order to develop field-applicable diagnostics for *Vgsc* CN, and as a prelude to investigating the possible association of CN with insecticide resistance, three assays were compared for their accuracy in CN estimation in this species. The gold standard method is droplet digital PCR (ddPCR), however, the hardware is prohibitively expensive for widespread utility. Here, ddPCR was compared to quantitative PCR (qPCR) and pyrosequencing. Across all platforms, CNV was detected in ≈10% of mosquitoes, corresponding to three or four copies (per diploid genome). ddPCR and qPCR-Std-curve yielded similar predictions for *Vgsc* CN, indicating that the qPCR protocol developed here can be applied as a diagnostic assay, facilitating monitoring of *Vgsc* CN in wild populations and the elucidation of association between the *Vgsc* CN and insecticide resistance.

## Introduction

The evolution of insecticide resistance in mosquitoes is typically associated with variation in the gene(s) encoding the insecticide target-site, or alterations in detoxification gene expression^1, 2^. Recently, studies have shown that gene duplication can also play an important role in the evolution of insecticide resistance^3–8^.

In mosquitoes, species of the *Culex pipiens* complex provide well-characterized examples of how CNVs can be associated with adaptations to insecticide pressure. For example, amplification of the carboxylesterase alleles A2, B2, A5 and B5 has been associated with resistance to organophosphates through elevated expression and insecticide detoxification^9, 10^. Additionally, duplication of the *Ace-1* locus has been linked to organophosphate and carbamate insecticide resistance in both *Culex pipiens* ^11^ and the malaria vector *Anopheles gambiae* ^12, 13^. There are fitness consequences for the *Ace-1* G119S mutation^14^. Indeed resistance management^15^ is predicated upon such fitness costs. The *Ace-1* duplication mitigates these costs^16^ through bringing a wild-type and resistant allele onto the same chromatid and is thought to partially compensate for the deleterious effects of resistant alleles in the absence of insecticide^17–20^.

Fitness costs for *Vgsc* mutations are less well studied. However, Platt *et al*.^21^ showed that *kdr* does indeed have fitness costs and the recent detection of duplicated *Vgsc* in Brazilian *Aedes* ^22^ is suggestive that duplication may be evolving at this locus in some mosquito populations, as for *Ace-1*, to counteract such negative effects.

*C*. *quinquefasciatus*, a mosquito with a broad distribution in tropical and subtropical regions is the main vector of lymphatic filariasis, West Nile virus (WNV) and St. Louis encephalitis virus (SLEV)^23, 24^. Recently, variation in the copy number of the *para*-type sodium channel gene (*Vgsc*) was described using Southern blot and PCR methods^25^ in field-collected Californian mosquitoes indicating that the *C*. *quinquefasciatus* genome may also contain at least a duplication of this gene.

Although CNVs have been described for both target-site and metabolic genes, and is associated with resistance to insecticides, readily applicable molecular diagnostic tests for CN are lacking although field applicable predictive assays are a goal for monitoring and tracking insecticide resistance^26^. Discovery methods for identifying CNVs such as microarrays and next-generation sequencing are laborious^27^, limiting the identification and development of diagnostic methods. Nevertheless, developing high-throughput, cost effective PCR-based approaches for large scale population genotyping are imperative for monitoring and elucidating the role of CNV in the evolution of resistance and on vector control.

The utility of CN assays will depend upon precision of CN estimation and assay cost. Low CN (e.g. single duplication) presents technical challenges for estimation of copy number, particularly where there is CNV within a population^28^. One promising assay for CN estimation is the droplet digital PCR (ddPCR) platform^29^ which has been shown to provide accurate quantification and high sensitivity across a range of CNs and sample types (e.g. refs 30–33). However, the high start-up cost of this platform precludes its utility in most field scenarios. In the mosquito *Anopheles gambiae* Djogbénou *et al*.^7^ have utilized ddPCR for accurate estimation of CN of *Ace-1* demonstrating fixed duplication of this gene in the studied populations. In the present study, the ddPCR assay was used as a gold-standard to score the number of copies of the *Vgsc* gene in field-collected *C*. *quinquefasciatus* from Uganda and the results compared to those from two quantitative PCR (qPCR) assays, and with pyrosequencing. We show that CNVs are detected in an average of 9.8% of the samples (across two or more genotyping platforms) but that the qPCR-Std-curve method utilized here yields similar predictions of *Vgsc* CN to ddPCR and is therefore an accurate, appropriate and affordable assay for determination of *Vgsc* CN.

## Results

### Vgsc gene haplotype diversity and genotype constitution

The potential for a gene duplication event in the *Vgsc* gene in Ugandan *C*. *quinquefasciatus* was suggested by abnormal TaqMan genotyping results for the *Vgsc*-L1014F mutation located in exon 20^34^. Two parallel assays for detecting the 1014F mutations were designed to genotype the wild type codon (TTA) and the two alternative resistant codons, (TTT or TTC), which both result in a pyrethroid and DDT resistance associated change from Leucine to Phenylalanine^35^.

**Figure 1:**
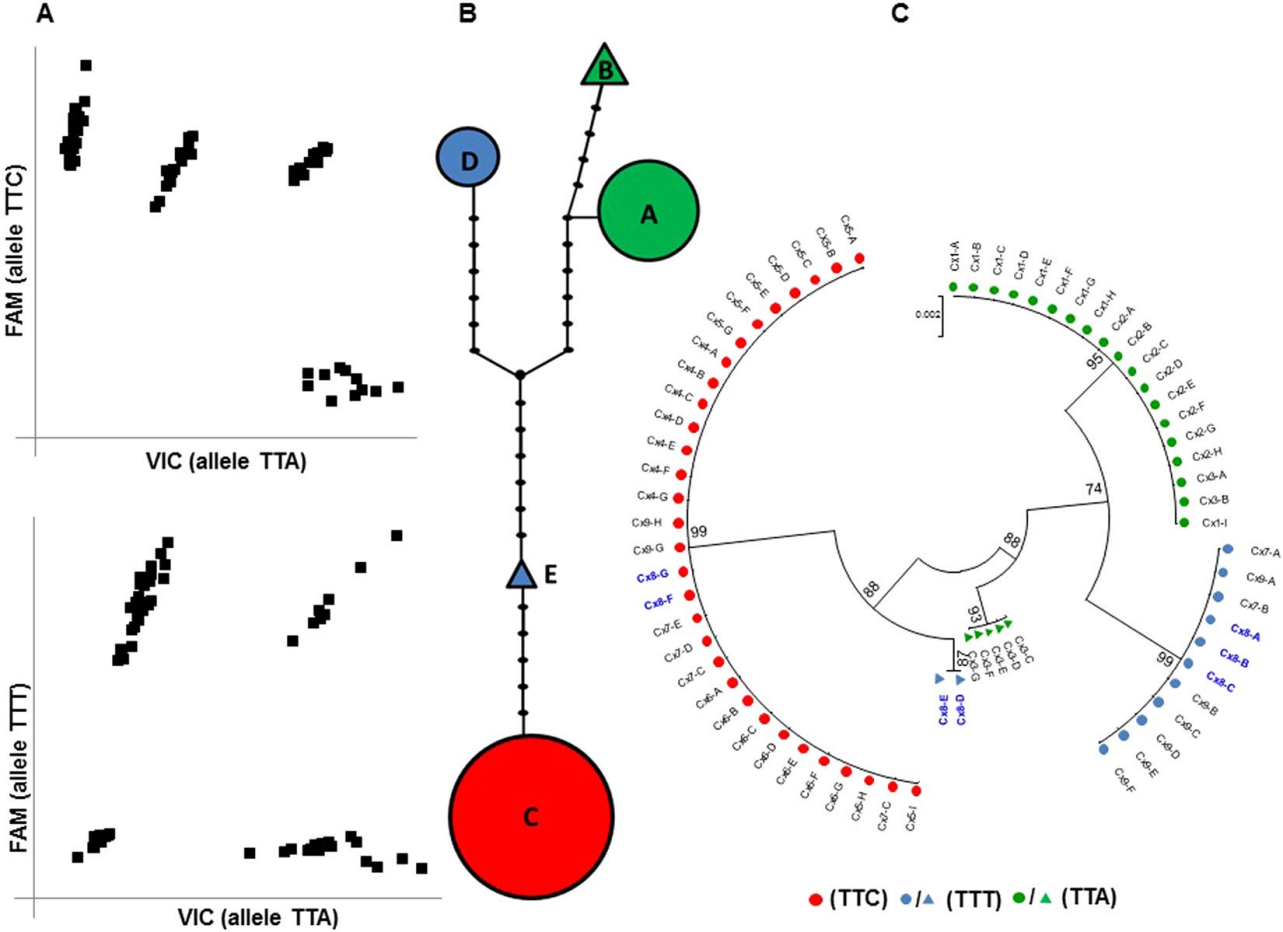
Haplotype diversity based on partial sequence of the *Vgsc* gene from Ugandan *C*. *quinquefasciatus* (**A**) Scatter plot of TaqMan-based allelic discrimination for TTA/TTC and TTA/TTT (where TTC and TTT represent alternative codons for the 1014F mutation). (**B**) Haplotype network of a 677 bp fragment of the *Vgsc* gene encompassing the 1014 codon. Polygon sizes denote the relative number of samples represented by each haplotype. The branches between black dots represent mutational steps separating observed haplotypes. (**C**) Dendrogram of individuals with a likely duplication of the *Vgsc* gene. (Cx number) represents an individual sample identifier and the letters A to I correspond to distinct colonies sequenced per sample. Individual Cx8 is highlighted in blue since this mosquito displayed ≥ two haplotypes. Green, red and orange shapes correspond to distinct *Vgsc-*1014 alleles.

The TaqMan assay employed two allele specific fluorogenic probes that produce a fluorescent signal proportional to the number of allele specific SNPs amplified in the qPCR reaction. In diploid individuals with single copy loci, individuals may be assigned to one of three clusters of fluorescence, one for each homozygous genotype and the other for heterozygotes. However, in this study genotyping of approximately 190 mosquitoes showed the presence of four well separated clusters instead of the normal three (Fig. 1A). We hypothesized that two putative heterozygous clusters may be due to gene duplication, which causes a shift in the fluorescence ratio of the two probes since individuals with CNV can possess >2 alleles.

To investigate if the TaqMan results were linked to variation in primer/probe sequence binding sites or due to variation in the number of alleles, we carried out a haplotype diversity analysis using 68 sequences from 12 individuals (GenBank accession numbers: KR061912-KR061979) of a 677 bp fragment of the *Vgsc* gene covering the Taqman primer/probe binding sites. Sequence analyses indicated the occurrence of five distinct haplotypes and 18 segregating sites plus seven gaps over all samples (Fig. 1B). Haplotype C, which displays the TTC 1014F mutation was the most common. The haplotype network suggested two origins for the TTT mutation due to the presence of fourteen mutations (nine SNPs and five polymorphic gaps) between haplotypes (Hap D and Hap E) bearing this mutation.

Haplotype analysis indicated no variation in the binding site of the primer/probes sequence in the mosquitoes studied (Figure S1) although did indicate that individual Cx8 possessed three distinct resistant haplotypes; two for the allele TTT and one for TTC (Fig. 1C), supporting our hypothesis that there is CNV in the *Vgsc* in the populations.

### Copy number assignment of the Vgsc gene based on PCR-methods

**Figure 2:**
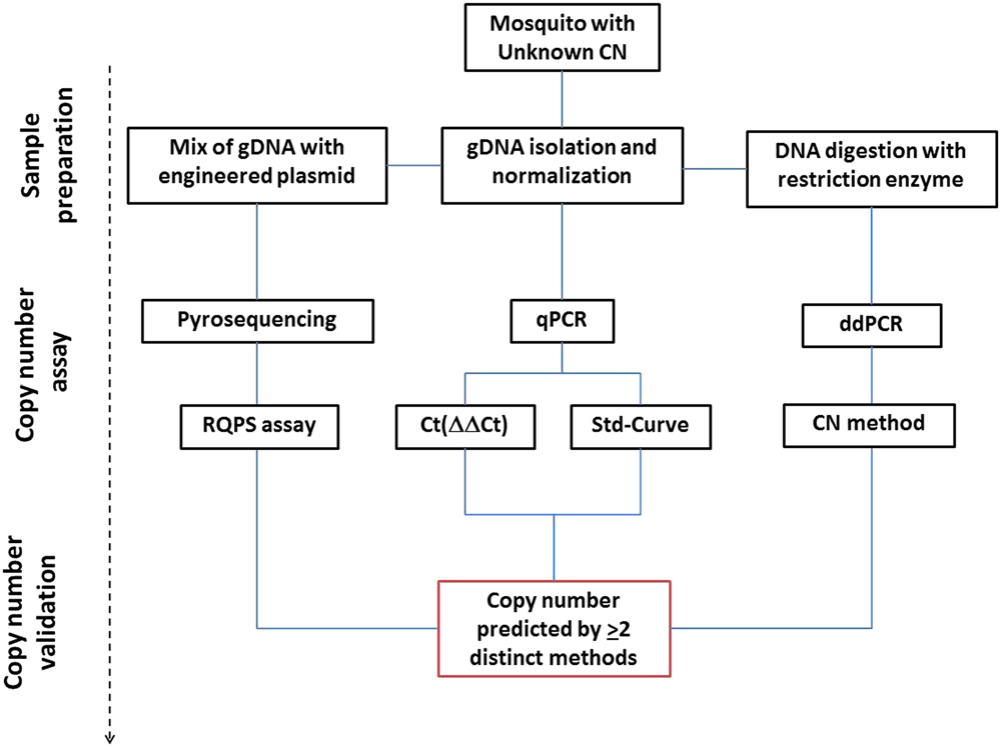
Schematic depicting the different approaches applied for genotyping and validation of the *Vgsc* gene CN in *C*. *quinquefasciatus* mosquitoes. qPCR-Std-curve and ddPCR-CN method applied an absolute quantification measurement, while qPCR-ΔΔCt and pyrosequencing-RQPS use a relative CN quantification. RQPS: Reference Query pyrosequencing. ddPCR: Droplet Digital PCR. Ct: intersection between an amplification curve and a threshold line.

The four PCR *Vgsc*-CN calculation methods utilised are described in Fig. 2. The CN assessment was conducted by normalizing the number of *Vgsc* gene copies to the *cAMP-dependent protein kinase A* (*Pka*) gene, a single copy housekeeping gene in the *Culex* genome (endogenous control).

### Vgsc copy number assessment by ddPCR

**Figure 3:**
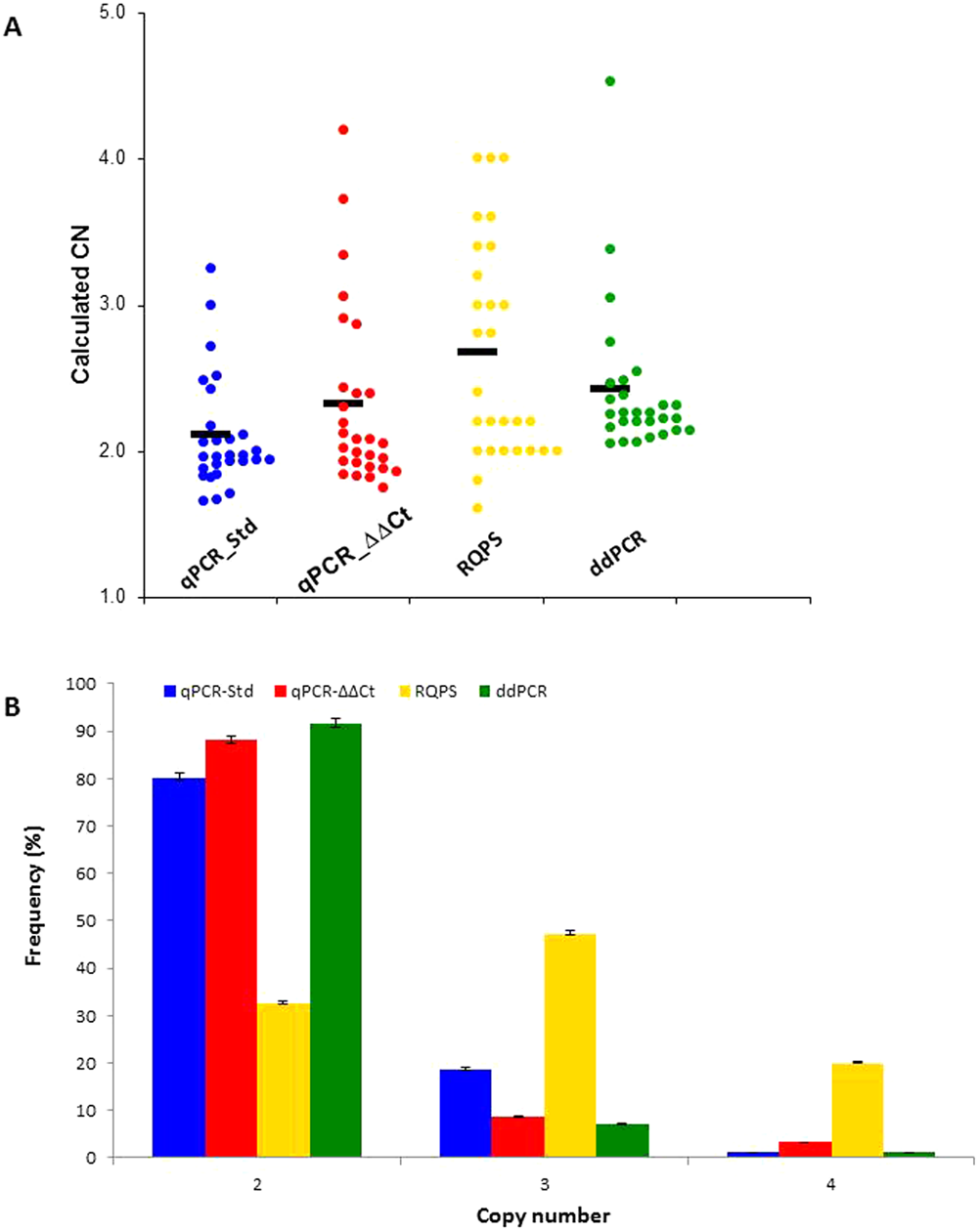
CN prediction across different methods. (**A**) Scatterplot of predicted CN per individual and genotyping method. Each dot represents the CN predicted for each individual, predicted CN corresponds to the calculated number without correction to expected CN. The black lines show the mean of predicted CN. (**B**) Direct comparison of the predicted CN frequency across different methods. Error bars show 95% confidence intervals.

The genotyping of 92 individuals from four Ugandan populations by ddPCR indicated the presence of CN ranging from 2–4 copies for the *Vgsc* gene per diploid genome (Fig. 3A). The mean number of droplets analysed for the replicate reactions varied between 13,860 and 24,821 droplets with the total number of FAM/VIC positive droplets no less than 10%. Between replicates, the 95% confidence interval of the calculated CN completely overlapped in most of the samples genotyped indicating strong reproducibility of the assay (Figure S2A). ddPCR results also indicated that the experimental conditions applied for the *Vgsc*-CN assay were efficient for complete separation of fluorescence amplitudes between positive and negative droplets as well as for identification of four distinct populations of droplets FAM+/+, VIC+/+, FAM/VIC+/− and FAM/VIC−/− (Figure S2B).

### Validation of the primer design for qPCR

Titration experiments with varying primer concentrations found that 400 nM of primers yielded the closest Ct value comparing reactions for the *Vgsc* and *Pka* gene based on SYBR-GREEN detection. 200 nM of probe was selected as this concentration yielded early Ct values that remained constant as compared to using higher concentrations of the probe (Figure S3). Primer efficiency for qPCR and digital droplet PCR (ddPCR) was evaluated using a standard curve performed on duplex PCR reactions run in triplicate for both genes. Amplification efficiency was similar for both *Vgsc* and *Pka* genes suggesting no evidence of reagent competition or primer/probe interactions, as indicated by PCR efficiencies of 98.7% and 101.9% respectively, and correlation coefficients of approx. 0.98 (Figure S4A). The specificity of the primer sets was verified on both agarose gel and melt curves, which showed single bands and melting peaks. Reproducibility of the duplex *Vgsc*-CN assay was confirmed by running three experiments on consecutive days indicating strong correlation between the first run and the second and the first and third, R^2^ = 0.879 and R^2^ = 0.980, respectively (Figure S4B).

### Vgsc copy number assessment by qPCR

For absolute quantification analysis by the qPCR-standard method, 92 samples were compared to a relative standard curve constructed with a plasmid encompassing a single copy of the *Pka* and *Vgsc* gene. The resulting standard curve was linear in the range tested, with a PCR efficiency close to 100% differing by only 2% between both genes (Figure S5). The standard curve covers Ct values ranging from 24 to 34, which interpolate a genomic DNA concentration around 10 ng/µl. The concentration of the *Vgsc* and *Pka* gene was determined from the relative standard curve in copies per microliter and was between 1115.65 and 939150.7 for the *Vgsc* gene and 31.25 and 669886.3 for the *Pka* gene. The ratio of *Vgsc*/*Pka* ranged from 0.8 to 1.70 with predicted CN between 2 and 4 (Fig. 3A).

The predicted CN as determined with the qPCR-ΔΔCt method indicated that individuals had 2–4 copies per diploid genome (Fig. 3B), with confidence intervals for the calculated CN higher than 0.99 in 78.9% of the individuals. The Ct values observed for the *Vgsc* and *Pka* gene ranged from 26.45 to 29.45 and 28.11 to 30.33, respectively with very little variation on the average Ct and a low standard deviation for the *Vgsc* (27.66, SD = 0.26) and *Pka* gene (28.85, SD = 0.23) across independent experiments.

### Vgsc copy number assessment by Reference Query Pyrosequencing (RQPS)

The assessment of the *Vgsc* CN using the *Vgsc*-RQPS method was carried out by comparing the peak ratio of the RQ-probe allele A, for *Vgsc* and for *Pka* in relation to the complementary gDNA alleles T and G using a linear regression where the intercept was set at zero. The CN of the samples was inferred by multiplying by 2 the slope of the linear regression (*y* = k*x*; where k is the slope) with assay quality verified using R^2^ value. In total, 92 samples were genotyped with a CN of 3 being the most frequent (in 47.3% of individuals) whereas CNs of 2 and 4 were observed in 33% and 20% of the samples (Fig. 3B). Application of a threshold of R^2^ ≥ 0.8 to assess the assay accuracy in the experimental conditions resulted in only 55.43% of the samples (N = 51) fitting the criterion. The other samples retained very low or negative R^2^ values, which indicated that the experimental conditions or assay design has low precision and reproducibility.

### Comparison between the calculated CN across the different methods

To compare the predicted CN of the *Vgsc* gene across the four different approaches the data set was reduced to the 51 individuals on which all assays were reliably conducted. The constraint was the number of samples genotyped by *Vgsc-*RQPS assay that fit the accuracy criterion described previously. For the combined data set including mosquitoes from all the study regions (Jinja, Kampala, Kanungu and Tororo) the predicted CN across the four methods ranged from 2 to 4 (Fig. 3B) with two copies (i.e. the standard diploid complement) being the most frequent CN observed by qPCR-ΔΔCt, qPCR-Std-curve and ddPCR (85.9%, 79.3% and 91.7%, respectively), whereas three copies was observed at higher frequency (47%) in mosquitoes genotyped using the QRPS method. The qPCR methods and ddPCR showed higher similarity of predicted CN distribution compared to RQPS (Fig. 3B), which in most of the cases overestimated the predicted CN by 1.

**Figure 4:**
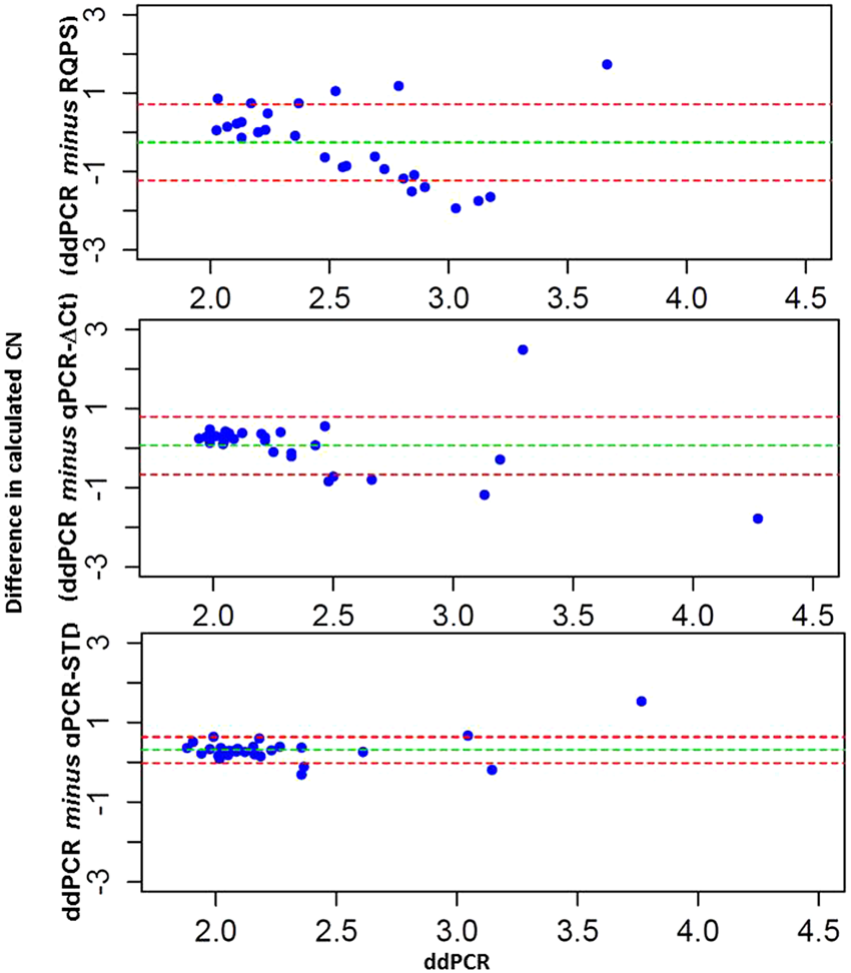
Bland-Altman plot showing the difference between the predicted CN of qPCR methods and RQPS against the ddPCR CN prediction. Each blue dot represents the CN difference for one individual. The dashed green line shows the mean difference, while the dashed red lines show 95% confidence limits.

Since analytical variation associated with PCR based methods such as amplification efficiencies between reactions can result in confounding results, validation of predicted CN is required to avoid misidentifying CNVs. Here we addressed this in two ways. Firstly, direct comparisons of the calculated CN between the ddPCR (assumed herein as the gold-standard standard due to the high precession described elsewhere^36^) and the other methods was conducted to identify discrepancies for posterior CN validation by re-genotyping. Using this approach, deviation was highest for *Vgsc*-RQPS and lowest for qPCR-Std-curve (Fig. 4). Indeed, only 6.5% of samples for which CN was calculated using the qPCR-Std-curve method were discrepant from the ddPCR results, whilst 28.3% of samples were discrepant for the qPCR-ΔΔCt method. Following re-genotyping, the predicted CN of these discrepancies increased by 1 copy, bringing them into line with the ddPCR CN and indicating initial genotyping inaccuracy of the qPCR methods, albeit at very low levels for the qPCR-Std-curve method. In contrast, since there was very large variation for most of the samples assayed by the RQPS method, 50% of the samples were randomly selected to be repeated, with no reduction in discrepancy compared to ddPCR prediction.

Secondly, to minimize the number of false positives; predicted CN from the different methods were merged into one final CN call for each sample. Using the criterion that the likely CN per individual should be identical for ≥2 CN methods, we identified that most individuals show no evidence of gene duplication, while in 9.8% of mosquitoes CN was detected. Crucially, we note that in those samples genotyped by ddPCR and in which duplication was identified, and for which we have corresponding results from Taqman genotyping (*N* = 6), all individuals display both TTA and TTC/TTT genotypes in the same individual suggesting that where CN exists it brings a 1014L- and 1014F-bearing copy together.

## Discussion

Resistance associated variation in the sodium channel gene (*Vgsc*), a target-site for pyrethroids and DDT insecticides, is a major threat to the success of control strategies for vector-borne diseases^37^. Various SNPs, or combination of SNPs, in the *Vgsc* gene, have been associated with a reduction in sensitivity of the *Vgsc* gene to insecticides^38–41^. Recently, duplication of the *Vgsc* gene has been detected in *A*. *aegypti* from Brazil^22^. Whether this duplication has any functional role in resistance remains to be tested, although duplication of genes such as *Ace-1*, and the resistance to dieldrin gene, *Rdl* ^13, 20, 42^ have already been demonstrated to be involved in insecticide resistance. Duplication, at least for *Ace-1* mitigates the inherent fitness costs of the mutation in the absence of insecticide and it is known that *Kdr* does have fitness costs^21^. Understanding the role of *Vgsc* CNV in the resistance phenotype requires accurate estimation of CN in individual samples. CN estimation is difficult at low CNVs^28^ since distinguishing two from three or four copies is problematical where there is variation in the quantitative nature of the signal. The problems of CN estimation are reduced where duplication is fixed in the population e.g. for *Ace-1* in *Anopheles* ^7^, where copy number is high in resistant insects due to gene amplification (e.g. the esterase B locus is amplified 30–250 times in Japanese and American populations of *Culex pipiens* respectively^43, 44^) or where crossing experiments allow greater control over the number of loci in parental strains and crosses^22^. In field-collected samples from populations in which CN varies it is imperative to have accurate, reliable and tested methodologies for CN estimation. Here we have utilized a range of methods on field-collected *Culex* samples. Pivotally, we establish CN in each sample first using the gold standard method of droplet digital PCR (ddPCR) which does not suffer from accuracy issues at low CN. However, the high cost of the hardware will likely be prohibitive for widespread use of this assay in most laboratories. We then show that a lower cost alternative is reliable, detects a similar population level of CNV and is implementable on equipment found in most standard molecular biology laboratories. The consistent CN prediction by both qPCR methods and ddPCR using the *Vgsc*-CN assay described herein thus provides more flexibility in assay choice for future studies – where ddPCR is available its inherent accuracy means that it is likely to be chosen. However, where ddPCR is not available (as in most laboratories) we have shown that the ddPCR and qPCR-Std-curve methods yield similar results for CN calculation with the lowest standard deviation. We note that both methods rely on absolute quantification, although calculated by different methods. Since the ddPCR platform is not broadly accessible due to high cost, our results indicate that the application of the qPCR-Std-curve method can provide a good alternative to precisely infer CNV. In contrast, the RQPS method shows the lowest concordance with other three methods. In addition, the RQPS calculated CN was much less precise. This may stem from issues with sample preparation since the RQPS method requires mixing of gDNA and RQPS-probe at 1:1 and 1:2 molar ratios, which increases sample manipulation steps and consequently can introduce more experimental errors. It is also possible that the overestimation of the CN by the RQPS method may be linked to inaccuracy in the mixing ratio of gDNA and the RQPS-probe since in about 50% of the samples the expected peak ratio of the gDNA and RQPS-probe alleles were not observed.

Our data focus on the sixth transmembrane segment of domain II of the *Vgsc* in which the known resistance-associated mutations occur, and this does not necessarily mean that the whole gene is duplicated. Xu *et al*.^25^ also report a duplication of the sodium channel, though they, like us, study only the region surrounding codon 1014. However, there is additional evidence that the complete sodium channel is duplicated in (African) *C*. *quinquefasciatus*. This gene is very poorly annotated in the *Culex* genome sequence^45^ partially as a consequence of the difficulties of the assembly within this genome^46^. However, if the complete *Culex* voltage-gated sodium channel sequence^34^ is used in a BLAST search of the *Culex* genome sequence (Johannesburg strain) it is evident that two voltage-gated sodium channel exist with one full length (exons 1–32) whilst for the other only exons 12–32 are evident (Table S1). It is not clear if this is indeed a partial gene sequence or a consequence of incomplete assembly (the exon hits are distributed across multiple contigs) though the latter seems extremely likely given the known assembly issues of this genome. Thus, we believe that the sodium channel duplication is potentially an old one (since it is found in the Johannesburg strain and in *C*. *quinquefasciatus* from America^25^) but our data demonstrate that this duplication is polymorphic, segregating in field populations in Uganda, and present in potentially >3 copies.

For *Ace-1* it is not copy number *per se* that is important for over-coming the deleterious fitness consequences of carrying the resistance allele, but instead the qualitative nature of having some acetylcholinesterase, bearing the wild-type 119G sequence (evolved to function in the absence of insecticide but targeted in the presence of insecticide), and some bearing the 119S mutation which has compromised function in the absence of insecticide but is under extremely strong selection in the presence of insecticide. Haplotypes with both a 119S allele and a 119G allele allow function when either insecticides are present or absent. It is likely that a similar situation exists for the *kdr* since it carries a fitness cost (e.g. in *Anopheles gambiae* ^21^ but also in some other arthropods^47^) and there is accumulating evidence from *Aedes* (e.g. ref. 22) that this is also true in this species. Consequently, a CNV where both a wild-type and ‘resistant’ allele occur, could be beneficial, so allowing maximally functioning sodium channel whether insecticide is present or absent. It seems that *Culex* may have been predisposed to develop a fitness cost ameliorating haplotype since the Johannesburg strain displays two copies of *Vgsc*. Our evidence from ddPCR/Taqman, suggests that where a duplication occurs, both a wild-type (TTA) and a ‘resistant’ (TTT or TTC) allele are typically found in the same individual. This provides evidence that this duplication has arisen to combat the fitness costs of the 1014F mutation.

The *Vgsc*-CN assays described here provides a simple and robust workflow for precise measurement of CN in field collected mosquitoes. Pan-genomic levels of CN variation in mosquitoes are currently unknown although we note that in the silkworm *Bombyx mori*, *in silico* analysis indicates that 1.4% of the genome is duplicated, including genes associated with immunity, detoxification and reproduction^48^. By replacing the primer/probe of the gene of interest in the approach used here, the method can be easily transferable to investigate the CN frequency of other genes displaying gene duplication in field collected *Culex* mosquitoes.

In summary, our data indicate the presence of CN variation in around 10% of the mosquitoes assayed, with variation in CN corresponding to three or four copies (diploid genome). Under our experimental conditions, the ddPCR and qPCR-Std-curve methods performed more precisely and yielded similar prediction of the *Vgsc* CN. The fact that where duplication is seen, both a 1014F and 1014L allele are often present in the same individual is indicative that this segregating CNV may have arisen to combat the fitness costs of resistance in the *Vgsc* ^21, 22^.

## Material and Methods

### Sample collection and DNA isolation

Indoor resting adult *C*. *quinquefasciatus* mosquitoes were collected from four sites in Uganda: Jinja, Kampala, Kanungu and Tororo between June and July 2012. Adults were sexed using antenna morphology with only males selected to characterize the *Vgsc* gene dose since gravidity in females can affect CN estimation. Samples were stored on silica gel prior to DNA isolation using a DNeasy kit (Qiagen) following the manufacturer’s recommendations. DNA concentration from each mosquito was quantified by PicoGreen (Life Technologies)^49^ and then normalized to approximately 10 ng/µl. Before CN analysis, all adult mosquitoes were confirmed as *C*. *quinquefasciatus* by a diagnostic PCR method^50^.

### L1014F-Vgsc allelic discrimination assays

Two assays to genotype the L1014F-*Vgsc* mutations in exon 20 of the *Vgsc* gene (see below), which has been implicated in resistance to pyrethroids and organochlorine insecticides were initially designed and applied in parallel to detect two non-synonymous variants; one to genotype TTA/TTT alleles and the other to detect TTA/TTC variants. Primer sets and TaqMan probes were designed using the Custom TaqMan® Assay Design Tool (Life Technologies).

TaqMan allelic discrimination reactions were carried out using approximately 20 ng of gDNA, 1x SensiMix II probe (Bioline), 0.4 µM of each primer (*Kdr*-F: 5′-CTTGGCCACCGTAGTGATAGG-3′ and *Kdr*-R: 5′-GCTGTTGGCGATGTTTTGACA-3′) and 0.1 µM of each probe (Probe-TTC-allele: 5′- FAM-CACGACGAAATTT-3′ or Probe-TTT-allele: 5′-FAM-TCACGACAAAATTT-3′ and Probe*-*TTA*-*allele/wildtype: 5′-VIC-ACTCACGACTAAATTT-3′), in a final volume of 10 µl. The PCR was performed on a Stratagene MX3005P with cycling parameters of 95 °C for 10 min followed by 40 cycles of 95 °C for 10 sec and 60 °C for 45 sec.

### Characterization of Vgsc haplotype diversity

To investigate the haplotype diversity and the number of distinct haplotypes present in each individual, a fragment of approximately 676 base pairs of the *Vgsc* gene spanning intron 19 and exon 20 including the position of the L1014F-*Vgsc* (*Kdr* mutation) originally described in houseflies^51^ and then other insects^52^ was sequenced. Identification of CN using haplotype diversity assumed that each individual mosquito carrying >2 distinct haplotypes exhibited copy number variation, as described by Labbé *et al*.^11^; however, if there is no variation between the gene copies the variation in CN could not be detected. The number of distinct haplotypes per individual was characterized by cloning and sequencing eight PCR clones per individual.

The partial fragment of the *Vgsc* gene was amplified by PCR in a reaction volume of 25 µl including approximately 25 ng of gDNA, 1x Phusion HF buffer, 200 µM of each dNTP, 0.02 U/µl of Phusion Hot start II DNA polymerase and 0.4 µM of each primer *Vgsc*-F: 5′-CCTCCCGGACAAGGACCTG-3′ and *Vgsc*-R: 5′-GGACGCAATCTGGCTTGTTA-3′. Amplification was performed with cycling conditions of 98 °C for 30 sec, followed by 30 cycles of 98 °C for 10 sec, 56 °C for 15 sec and 72 °C for 15 sec. with a final extension of 72 °C for 10 min. PCR products were purified using the GeneJet PCR purification kit (Thermo Scientific) and cloned into the pJet 1.2 vector using the CloneJet PCR cloning kit (Thermo Scientific). Individual plasmids were isolated using the GeneJet Plasmid Mini Kit and sequenced (Source Biosciences).

Sequence traces were edited in CodonCode Aligner software version 4.2.2. Multiple sequence alignments were performed with ClustalW and then visualized using Jalview software^53^. Haplotype diversity was visualized using a Neighbour-Joining tree build using the software MEGA 5.1^54^ with frequency and relationships between haplotypes visualized by a haplotype network generated using the program TCS version 1.21 treating gaps as a fifth character^55^.

### Vgsc gene CN assignment by PCR-based assay

The *Vgsc*-CN PCR-based methods described here were designed to perform on three platforms using four distinct CN calculation methods (Fig. 2). The CN assessment is based on a partial fragment of exon 20 of the *Vgsc* gene (CPIJ007595-RA) normalized to a fragment of exon 1 of the *cAMP-dependent protein kinase A* (*Pka*) gene (CPIJ018257-RA), a single copy housekeeping gene in the *Culex* genome (endogenous control).

The assays based on real-time and ddPCR platforms employed a TaqMan-CNV method, which consists of a duplex PCR reaction using a pair of unlabeled primers for each gene and a FAM-MGB probe for the *Vgsc* gene and a VIC-MGB probe for the reference gene (*Pka*). The CN quantification by pyrosequencing was conducted using the Reference Query Pyrosequencing (RQPS) method described by Liu *et al*.^56^ with minor modifications. Briefly, the *Vgsc*-RQPS method utilizes an engineered plasmid (probe) encompassing a 100 bp fragment from both the *Vgsc* and *Pka* genes linked to any gene fragment (stuffer DNA – in this case we used a fragment of the actin gene CPIJ012573, see Supplementary methods) with no homology to the reference or query gene. On each fragment a SNP was introduced that differed between the RQ-probe allele and the gDNA allele. gDNA of each mosquito with unknown CN was mixed with the RQ-probe and then co-amplified in a simplex PCR reaction for each *Vgsc* and *Pka* gene followed by pyrosequencing analysis.

### Vgsc-CN primer design and validation

All primer and probe binding sites for exon 20 of the *Vgsc* gene were selected using the sequence alignment from the haplotype diversity analysis to identify conserved regions (Figure S1). *Vgsc*-CN assay primers and probes used on the qPCR and ddPCR assay were designed using the primer express version 2.0 Software (Applied Biosystems). Primers and probes for the *Vgsc* gene were: *Vgsc*/CN-F: 5′-TGCCACGGTGGAACTTCA-3′; *Vgsc*/CN-R: 5′-CACCCGGAACACGATCATG-3′; *Vgsc*/CN-Probe: 5′-FAM-GACTTCATGCACTCAT-MGB-3′, while for the *Pka* reference were: *PKA*/CN-F: 5′-GACTGGTGGGCATTAGGTGTTC-3′; *PKA*/CN-R: 5′-TCAGCAAAAAAAGGTGGATATCC-3′; Probe: 5′-VIC-GTGTACGAGATGGCAGC-MGB-3′.

For the pyrosequencing assay, PCR primer sets and sequencing primers that co-amplify the genomic and RQ-probe sequences for both *Vgsc* and the reference gene were designed using the PyroMark assay design software 2.0 (Qiagen). For the *Vgsc* gene, PCR reactions were performed using the primers: *Vgsc*/Py-F: 5′-CGAATCCATGTGGGACTGC–3′ and *Vgsc*/Py-R: 5′Biotin- CTATCACTACGGTGGCCAAGAAGA-3′, whereas for the *Pka* gene the primers used were: *PKA*/Py-F: 5′*-*GGAAACAACGCAACTTCAACA-3′ and *PKA*/Py-R: 5′Biotin- TCTTCTTTAGCTTGATCCAGGAAT-3′.

The efficiency of primers and probes designed for qPCR and ddPCR were determined by using a standard curve for three replicates across five doubling dilutions from an initial concentration of approximately 20 ng/µl of gDNA. Primer specificity was tested by melt curve and electrophoresis on a 2% agarose gel. Duplex-PCR reaction conditions were experimentally determined by primer-limiting analysis to identify the optimal primer and probe concentrations that provide a constant Ct value (threshold cycle) among primer/probes titration with primer efficiency on duplex-PCR reaction not differing by more than 5%.

### Copy number assignment using qPCR

Absolute and relative quantification methods were used in parallel to quantify the *Vgsc* CN. For both quantification methods qPCR reactions were performed in triplicate in a final volume of 20 µl including around 10 ng of genomic DNA, 1x TaqMan gene expression master mix (Applied Biosystems), 0.4 µM and 0.2 µM of each primer and probe as described previously. Two samples assayed earlier were used as positive controls of PCR reproducibility. Amplification was conducted using the Applied Biosystems 7500 Fast PCR-Real time systems with conditions of 50 °C for 2 min, 95 °C for 10 min, and then 40 cycles of 94 °C for 15 s and 60 °C for 1 min.

For absolute quantification, a plasmid containing the sequences spanning primer and probe binding sites for both genes used in the qPCR assay was created (supplementary methods). The purified plasmid concentration was measured using picogreen and then a 10-fold serial dilution ranging from 3 × 10^5^ to 10^1^ copies/µl of the *Vgsc*-*Pka* plasmid DNA was used to generate standard curves by plotting C_t_ values versus log copies for both *Vgsc* and *Pka*. Absolute copy number was calculated by determining the number of *Vgsc* and *Pka* copies per haploid genome interpolated from the standard curve for each sample and then the ratio (*Vgsc*/*Pka*) of copies/µl was multiplied by two to obtain the diploid genome CN. To increase the precision of the quantification, plates were used where the standard curve had R^2^ ≥ 0.98. The relative quantification between the *Vgsc* and *Pka* gene was assessed based on Ct values collected using a 0.2 threshold and automatic baseline. The CN analysis was carried out using the CopyCaller software v2.0 (Applied Bisystems), which applies a comparative (ΔΔCt) method.

### Copy number assignment by ddPCR

For the ddPCR assay, roughly 10 ng of gDNA was digested with 0.2 units of *Alu*I (NEB) for 15 min at 25 °C. *Alu*I was selected since its restriction sites were identified nearby the upstream and downstream position of the PCR primers for both the *Vgsc* and reference gene. Digested gDNA was assayed in a duplex ddPCR reaction in a final volume of 20 µl containing 1x ddPCR supermix, 0.4 µM of each primer and 0.2 µM of each probe. The total volume of each ddPCR PCR mix was transferred to the sample wells on the eight-channel droplet generator cartridge (Bio-Rad) while 70 µl of droplet generation oil (Bio-Rad) were loaded on each oil well channel. Finally, 40 µl of the partitioned droplet PCR mix were transferred to a 96-well plate and then amplified to end point using a thermal cycler.

The amplification conditions were determined by serial dilution of the *Vgsc*-*Pka* plasmid DNA to identify the required input gDNA concentration, while a temperature gradient ranging from 55 °C to 65 °C was conducted to detect assay amplitude with a well-defined separation between positive and negative droplet populations (Figure S6). Thermal cycling conditions were: 95 °C for 5 min, 95 °C for 30 sec and 57 °C for 1 min (40 cycles) and 98 °C for 10 min.

After PCR amplification, the PCR product was loaded on the QX100 droplet reader (Bio-Rad), for simultaneous two-colour detection of the droplets. Data analysis of the ddPCR reads was carried out using QuantaSoft analysis software version 1.6.6 (Bio-Rad). Absolute quantification of the *Vgsc* gene CN for each sample was then calculated in relation to the *Pka* gene event number.

### Copy number assignment using Pyrosequencing

Relative quantification analysis by the *Vgsc*-RQPS method required the construction of a plasmid (termed the RQ-probe), which contained partial sequences of the *Vgsc* and *Pka* gene with an introduced SNP for differentiating RQ-probe alleles from gDNA alleles. The RQ-probe design, cloning and purification details are described in the Supplementary methods.

For each sample tested, two mixtures of RQ-probe/gDNA were prepared using molar ratios of 1:1 and 2:1 in a final volume of 10 µl. Simplex PCR reactions for the *Vgsc* and *Pka* gene were performed in a total of 25 µl using 3 µl of each RQ-probe/gDNA molar ratio mix in parallel, 200 µM of each dNTP, 1x PCR buffer, 2.0 mM of MgCl_2_, 0.6 units of HotStarTaq DNA polymerase (Qiagen) and 0.4 µM of each primer. After initial denaturation at 95 °C for 15 min, PCR was performed for 40 cycles of 94 °C for 30 sec, 58 °C for 30 sec, and 72 °C for 30 sec, followed by a final extension step at 72 °C for 10 min.

Single-stranded PCR products for analysis by pyrosequencing were obtained using the PyroMark Q24 Vacuum Prep Workstation. Pyrosequencing reactions of the *Vgsc* PCR products were performed using the sequencing primer: 5′-TGCTGGTGGGCGACG-3′ and dispensation order: 5′-GTGATCTG-3′, whereas for the *Pka* PCR, amplification used the sequencing primer: 5′-CCGCAGAAAGTGTAAAA-3′ and the following dispensation order: 5′- TCGATCTG-3′. Pyrosequencing reactions were performed using the PyroMark Gold Q96 reagent kit (Qiagen) following the manufacturer’s guidelines.

CN prediction was calculated comparing the amplification ratios of the *Pka* reference gene (RQprobe-*Pka*/gDNA-*Pka* alleles) and *Vgsc* (RQprobe-*Vgsc*/gDNA-*Vgsc* alleles) by linear regression, with differences of the amplification ratios reflecting variation in gene copy number. The linear regression for the slope of the curve was multiplied by two to acquire the predicted CN in the diploid genome. Further details of the data analysis are described by Liu *et al*.^56^.

## Electronic supplementary material

Supplementary Information
