# Supplementary material for: Detection and quantitation of copy number variation in the voltage-gated sodium channel gene of the mosquito Culex quinquefasciatus
Source: Sci Rep. 2017 Jul 19;7:5821. doi: 10.1038/s41598-017-06080-8 (PMC5517494; doi:10.1038/s41598-017-06080-8)
Supplement: Supplementary file 1 — Supplementary Information [file 41598_2017_6080_MOESM1_ESM.pdf]

Supplementary Information to:

Detection and quantitation of copy number variation in the voltage-gated sodium channel gene of the mosquito *Culex quinquefasciatus*

Walter Fabricio Silva Martins[1,2], Krishanthi Subramaniam[1], Keith Steen[1], Henry Mawejje[3], Triantafillos Liloglou[4], Martin James Donnelly[1,5], Craig Stephen Wilding [6]

[1] Department of Vector Biology, Liverpool School of Tropical Medicine, Liverpool, UK, [2] *Departamento de Biologia, Universidade Estadual da Paraíba, Campina Grande, Brasil,* [3] Infectious Diseases Research Collaboration, Kampala, Uganda, [4] Department of Molecular and Clinical Cancer Medicine, Roy Castle Lung Cancer Research, *Liverpool, UK,* [5] Malaria Programme, Wellcome Trust Sanger Institute, Cambridge, UK., [6] *Liverpool John Moores University, Liverpool, UK.*

Supplementary Methods

1. RQPS Reference-query probe design and construction

The RQ-probe design for *C. quinquefasciatus* Nav CNV analysis was constructed by the fusion of a partial fragment of the exon 1 of the *PKA* gene (reference single copy gene in the *Culex* genome) and of exon 20 of the *Nav* gene to a fragment of 386 bp of the *Actin* gene (CPIJ012573) used as stuffer DNA (Figure SM1). A SNP that differentiates the RQ-probe allele construct from the gDNA allele was introduced on the selected fragment of each gene as shown in Figure SM1.

The RQ plasmid construction was executed through two rounds of PCR. The first step was conducted in a 50 µl reaction containing approximately 40 ng of genomic DNA, 1x Phusion HF buffer, 200 µM of each dNTP, 0.4 µM of each primer Cx.ref-FI and Cx.quer-RI (Table SM1, Figure SM2) and 0.02 U/µl of Phusion DNA polymerase. Cycling conditions were 98 °C for 30 sec, 25 cycles of 98 °C for 10 sec, 58 °C for 30 sec and 72 °C for 15 sec, with a final extension of 72 °C for 5 min. The second round of the fusion PCR was carried out using the same concentration and conditions of the first, with the exception of the genomic DNA and primers, which were replaced by the product of the first reaction diluted 1:10 and the primers Cx.ref-FII and Cx.quer-RII, respectively.

The PCR product from the second round of the fusion PCR was loaded on a 2% agarose gel, and then the 551 bp fused fragment (Figure SM2) was extracted, purified and cloned into the pJet1.2 PCR vector (Thermo Scientific). Finally, after the plasmid had been purified and sequenced to confirm the correct insertion of the chimeric QR-probe in the vector, it was linearized with *Alu*I.

A)

5`–ATGGGAAACAACGCAACTTCAACAAATAAAAAAGTAGATGCCGCAGAAAGTGTAA
AA**S**AATTCCTGGATCAAGCTAAAGAAGATT–3`

B)

5`–GATCGAATCCATGTGGGACTGCATGCTGGTGGGCGACGTG**W**CCTGCATTCCGTTCT
TCTTGGCCACCGTAGTGATAGGAA–3`

C)

5`–TGGTATCCTCACCCTGAAGTACCCGATCGAGCACGGTATCATCACCAACTGGGATGA
TATGGAGAAGATCTGGCATCACACCTTCTACAATGAGCTGCGTGTTGCCCCAGAGGAGC
ACCCAGTCCTGCTGACTGAGGCCCCCCTGAACCCCAAGGCTAACCGCGAGAAGATGAC
TCAGATCATGTTTGAGACCTTCAACTCGCCAGCCATGTATGTTGCCATCCAGGCTGTCCT
GTCCCTGTACGCTTCCGGTCGTACCACCGGTATCGTTCTGGATTCCGGAGATGGTGTCTC
TCACACCGTCCCAATCTATGAAGGTTATGCTCTGCCCCATGCCATCCTCCGTCTGGATCT
GGCTGGTCGCGATCTGACCGACTACCTGATGA–3`

Figure SM1. Sequences of genomic DNA used for construction of the RQPS probe. A and B) partial fragments of the *Pka* and *Vgsc* gene respectively, with bold and underlined bases corresponding to a SNP introduced to differentiate RQ-alleles from gDNA sequence. C) Partial fragment of the *Actin* gene (CPIJ012573) used as stuffer region. S; G→C. W, T→A.

A)

Step 2

5`–ATGGGAAACAACGCAACTTCAACAAATAAAAAAGTAGATGCCGCAGAAAGTGTAAAA**C**AATTCCT

Step 1

GGATCAAGCTAAAGAAGATT*TGGTATCCTCACCCTGAAGTACCCGATCGAGCACGGTATCATCACCAACTG*

GGATGATATGGAGAAGATCTGGCATCACACCTTCTACAATGAGCTGCGTGTTGCCCCAGAGGAGCACCCAGT

CCTGCTGACTGAGGCCCCCCTGAACCCCAAGGCTAACCGCGAGAAGATGACTCAGATCATGTTTGAGACCTT

CAACTCGCCAGCCATGTATGTTGCCATCCAGGCTGTCCTGTCCCTGTACGCTTCCGGTCGTACCACCGGTAT

CGTTCTGGATTCCGGAGATGGTGTCTCTCACACCGTCCCAATCTATGAAGGTTATGCTCTGCCCCATGCCATC

Step 1

*CTCCGTCTGGATCTGGCTGGTCGCGATCTGACCGACTACCTGATGA*GATCGAATCCATGTGGGACTGCATG

Step 2

CTGGTGGGCGACGTG**A**CCTGCATTCCGTTCTTCTTGGCCACCGTAGTGATAGGAA–3`

B)

Pka- Forward | *Pka*-sequencing primer | *Pka*- Reverse

5`–ATGGGAAACAACGCAACTTCAACAAATAAAAAAGTAGATGCCGCAGAAAGTGTAAAA***S****AA*TTCCTGG

ATCAAGCTAAAGAAGA*TTTGGTATCCTCACCCTGAAGTACCCGATCGAGCACGGTATCATCACCAACTGG*

GATGATATGGAGAAGATCTGGCACCACACCTTCTACAAYGAGCTGCGTGTKGCCCCRGAGGAGCACCCAG

TCCTGCTGACTGAGGCCCCCCTSAACCCSAAGGCTAACCGCGAGAAGATGACTCAGATCATGTTTGAGAC

CTTCAACTCGCCRGCCATGTAYGTCGCCATCCAGGCTGTCCTGTCCCTGTACGCTTCCGGTCGTACCACC

GGTATCGTTCTGGATTCCGGAGATGGTGTCTCTCACACCGTCCCAATCTATGAAGGTTATGCYCTGCCCC

Nav- Forward

*ATGCCATCCTCCGTCTGGATCTGGCTGGTCGCGATCTGACCGACTACCTGATGA*GATCGAATCCATGTGG

Nav-Sequencing primer | *Nav* -Reverse

GACTGCATGCTGGTGGGCGACGTG***W***CCTGCATTCCGTTCTTCTTGGCCACCGTAGTGATAGGAA-3`

Figure SM2. Fusion PCR and pyrosequencing primer locations on the RQ-plasmid. A) Position of both primer sets used in the fusion PCR. Sequences in red and blue font correspond to partial sequences of the *Pka* and *Vgsc*, respectively. Sequence in green corresponds to the partial fragment of the Actin gene used to link both *Vgsc* and *Pka* B) Sequence of the RQ-plasmid probe showing the location of the SNP introduced in both *Pka* (S; G to C) and *Vgsc* (W, T to A) and PCR and sequencing primers annealing positions used for the RQPS method.

Table SM1. Primers for RQ plasmid construction by fusion PCR**.**

Primer	Sequence 5`-3`
	**Fusion PCR Step I**
Cx.ref-FI	GATGCCGCAGAAAGTGTAAAACAATTCCTGGATCAAGCTAAAGAAGA**TT**TGGTATCCTCACCCTGAAGT
Cx.quer-RI	AGGTCACGTCGCCCACCAGCATGCAGTCCCACATGGATTCGATCTCATCAGGTAGTCGGTCAGAT
	**Fusion PCR Step II**
Cx.ref-FII	ATGGGAAACAACGCAACTTCAACAAATAAAAAAGTAGATGCCGCAGAAAGTGTAAAACA
Cx.quer-RII	GAATTCTTCCTATCACTACGGTGGCCAAGAAGAACGGAATGCAGGtCACGTCGCCCACCAG

Construction of the *Vgsc-Pka* plasmid standard for qPCR absolute quantification

The plasmid used for construction of the standard curve for qPCR absolute quantification was developed by the fusion of partial fragments of the *Vgsc* and *Pka* gene including the PCR and probe region of the Nav-CN assay. The fusion PCR reaction was carried out in two steps. The first round was conducted in a final volume of 50 µl containing approximately 40 ng of gDNA, 1x Phusion HF buffer, 200 µM of each dNTP, 0.4 µM of each primer (Cx.*Vgsc-Pka*-P1: 5'-GCAAAGGATATAACAAAGCAGT-3' and Cx.*Vgsc-Pka*-P2: 5'-AAACCATCTATGCCCTTTGATCTAAGTAGATCCTTTAGTTCTGAC-3') and 0.02 U/µl of Phusion DNA polymerase. For the second step, the same conditions as the first step were used with the first PCR product diluted 1:10 as template and the external primers (Cx.*Vgsc-Pka*-P3: 5'-TCAAAGGGCATAGATGGTTTACAACGTGGACCGCTTC-3' and Cx. *Vgsc-Pka*-P4: 5'-TGCAGTCCCACATGGATTC-3') used. Both steps were amplified with the following conditions: 98 °C for 30 sec, 25 cycles of 98 °C for 10 sec, 57 °C for 30 sec and 72 °C for 15 sec, with a final extension of 72 °C for 5 min. Finally, the plasmid containing the *Vgsc-Pka*

fragment of 488 bp (Figure SM2) was linearized with *Alu*I followed by purification of the digested product extracted from a 2% agarose gel.

5`–CTATGTGGAACTCCAGAATATTTGGCACCAGAAATAATTTTAAGCAAAG
GATATAACAAAGCAGTT*GACTGGTGGGCATTAGGTGTTC*TTGTGTACGAGATG
GCAGCC*GGATATCCACCTTTTTTTGCTGA*TCAGCCAATACAAATTTATGAAAA
AATTGTTTCAGGAAAGGTACGATTTCCATCTCATTTCGGGTCAGAACTAAAG
GATCTACTTAGAAATCTTCTACAAGTTGATTTAACAAAACGTTACGGAAATC
TAAAAGCAGGAGTTAACGACATCAAAGGGCATAGATGGTTTACAACGTGGA
CCGCTTCCCGGACAAGGACC*TGCCACGGTGGAACTTCA*CCGACTTCATGCACT
CATT*CATGATCGTGTTCCGGGTG*CTGTGCGGCGAGTGGATCGAATCCATGTGG
GACTGCATGCTGGTGGGCGACGTGTCCTGCATTCCGTTCTTCTTGGCCACCG
TAGTGATAGGAAATTTAGTC–3`

Figure SM3. Partial sequence of the *Vgsc-Pka* plasmid, which corresponds to the linked partial fragments of the *Vgsc* and *Pka* gene obtained by fusion PCR

Supplementary Results

```
Cx.1/1-676     1 .......................................................... 58
Cx.2/1-676     1 .......................................................... 58
Cx.3/1-677     1 .......................................................... 58
Cx.4/1-677     1 .......................................................... 58
Cx.5/1-677     1 .......................................................... 58
Cx.6/1-677     1 .......................................................... 58
Cx.7/1-677     1 .......................................................... 58
Cx.8/1-677     1 .......................................................... 58
Cx.9/1-677     1 .......................................................... 58
    Consensus    GGACGCAATCTGGCTTGTTAACTTGTTTTTCACGAACTTGAGCGCGGCCGCGATGTTC

Cx.1/1-676    59 .......................................................... 116
Cx.2/1-676    59 .......................................................... 116
Cx.3/1-677    59 .......................................................... 116
Cx.4/1-677    59 .......................................................... 116
Cx.5/1-677    59 .......................................................... 116
Cx.6/1-677    59 .......................................................... 116
Cx.7/1-677    59 .......................................................... 116
Cx.8/1-677    59 .......................................................... 116
Cx.9/1-677    59 .......................................................... 116
    Consensus    GCCTTGATCCAGTTGGAGAAGCGCGATATCCGGTTGAACGCCTCGGCGATCTTGTTCG

Cx.1/1-676   117 .......................................................... 174
Cx.2/1-676   117 .......................................................... 174
Cx.3/1-677   117 .......................................................... 174
Cx.4/1-677   117 .......................................................... 174
Cx.5/1-677   117 .......................................................... 174
Cx.6/1-677   117 .......................................................... 174
Cx.7/1-677   117 .......................................................... 174
Cx.8/1-677   117 .......................................................... 174
Cx.9/1-677   117 .......................................................... 174
    Consensus    TTTCGTTGTCGGCTGTGGGCGCCGACAAACTCGAGGAACCAAAGTTGGACAAAAGCAA

Cx.1/1-676   175 ...................................AG..................... 232
Cx.2/1-676   175 ...................................AG..................... 232
Cx.3/1-677   175 .......................................................... 232
Cx.4/1-677   175 .......................................................... 232
Cx.5/1-677   175 .......................................................... 232
Cx.6/1-677   175 .......................................................... 232
Cx.7/1-677   175 .......................................................... 232
Cx.8/1-677   175 .......................................................... 232
Cx.9/1-677   175 .......................................................... 232
    Consensus    GGCTAAGAAAAGGTTAAGAACCTGGTTTCGGGAGGGAAGAGAAGATCGTTGTCAGTGG

Cx.1/1-676   233 G..............................-----...................... 285
Cx.2/1-676   233 G..............................-----...................... 285
Cx.3/1-677   233 ...............................-----...................... 285
Cx.4/1-677   233 ...............................-----...................... 285
Cx.5/1-677   233 ...............................-----...................... 285
Cx.6/1-677   233 ...............................-----...................... 285
Cx.7/1-677   233 ...............................-----...................... 285
Cx.8/1-677   233 ...............................-----...................... 285
Cx.9/1-677   233 ...............................-----...................... 285
    Consensus    AGGGTAGGCTCGCATGCGAGATTCCAATCGC-----GGAGCTGTCAAAACAACAACCA

Cx.1/1-676   286 ..................................A-.T.................... 342
Cx.2/1-676   286 ..................................A-.T.................... 342
Cx.3/1-677   286 ...................................--..................... 341
Cx.4/1-677   286 ...................................--..................... 341
Cx.5/1-677   286 ...................................--..................... 341
Cx.6/1-677   286 ...................................--..................... 341
Cx.7/1-677   286 ...................................--..................... 341
Cx.8/1-677   286 ...................................--..................... 341
Cx.9/1-677   286 ...................................T...................... 343
    Consensus    AGACAATTGCACCTTTAGGTGTGGACCTTCATCTG-AATTTTTTTTAAAGAACGCAAC
```

```
Cx.1/1-676   343 .........A.........G.--................................... 398
Cx.2/1-676   343 .........A.........G.--................................... 398
Cx.3/1-677   342 .......................................................... 399
Cx.4/1-677   342 .......................................................... 399
Cx.5/1-677   342 .......................................................... 399
Cx.6/1-677   342 .......................................................... 399
Cx.7/1-677   342 .......................................................... 399
Cx.8/1-677   342 .......................................................... 399
Cx.9/1-677   344 .........A.........G.--................................... 399
     Consensus   CTTAAAGAATTATTTACTATAGTATGATCGGTATGAACTGTTTGTTTACATCAAAGGT

Cx.1/1-676   399 .......................................................... 456
Cx.2/1-676   399 .......................................................... 456
Cx.3/1-677   400 .......................................................... 457
Cx.4/1-677   400 .......................................................... 457
Cx.5/1-677   400 .......................................................... 457
Cx.6/1-677   400 .......................................................... 457
Cx.7/1-677   400 .......................................................... 457
Cx.8/1-677   400 .......................................................... 457
Cx.9/1-677   400 .......................................................... 457
     Consensus   ACAAATGTGACCTTAAAGTTTTTCGTTCCCACCTTTTCTTGCATGCTGTTGGCGATGT

Cx.1/1-676   457 .............GC..................G.T...................... 514
Cx.2/1-676   457 .............GC..................G.T...................... 514
Cx.3/1-677   458 .......................................................... 515
Cx.4/1-677   458 .......................................................... 515
Cx.5/1-677   458 .......................................................... 515
Cx.6/1-677   458 .......................................................... 515
Cx.7/1-677   458 .......................................................... 515
Cx.8/1-677   458 .......................................................... 515
Cx.9/1-677   458 .............GC..................G.T...................... 515
     Consensus   TTTGACAGCTCTAATGCGCACACTAGATCAATCACGAAGACTTCACGCTGGAATACTC

Cx.1/1-676   515 .....T.................................................... 572
Cx.2/1-676   515 .....T.................................................... 572
Cx.3/1-677   516 .......................................................... 573
Cx.4/1-677   516 .......................................................... 573
Cx.5/1-677   516 .......................................................... 573
Cx.6/1-677   516 .......................................................... 573
Cx.7/1-677   516 .......................................................... 573
Cx.8/1-677   516 .......................................................... 573
Cx.9/1-677   516 .....T.................................................... 573
     Consensus   ACGACGAAATTTCCTATCACTACGGTGGCCAAGAAGAACGGAATGCAGGACACGTCGC

Cx.1/1-676   573 .....................................A.................... 630
Cx.2/1-676   573 .....................................A.................... 630
Cx.3/1-677   574 .......................................................... 631
Cx.4/1-677   574 .......................................................... 631
Cx.5/1-677   574 .......................................................... 631
Cx.6/1-677   574 .......................................................... 631
Cx.7/1-677   574 .......................................................... 631
Cx.8/1-677   574 .......................................................... 631
Cx.9/1-677   574 .....................................A.................... 631
     Consensus   CCACCAGCATGCAGTCCCACATGGATTCGATCCACTCGCCGCACAGCACCCGGAACAC

Cx.1/1-676   631 ..............................................             676
Cx.2/1-676   631 ..............................................             676
Cx.3/1-677   632 ..............................................             677
Cx.4/1-677   632 ..............................................             677
Cx.5/1-677   632 ..............................................             677
Cx.6/1-677   632 ..............................................             677
Cx.7/1-677   632 ..............................................             677
Cx.8/1-677   632 ..............................................             677
Cx.9/1-677   632 ..............................................             677
     Consensus   GATCATGAATGAGTGCATGAAGTCGGTGAAGTTCCACCGTGGCAGG
```

Figure S1. Multiple sequence alignment of a partial fragment of the *Vgsc* gene. Alignment was performed using ClustalW. Underlined regions in blue and black correspond to primer and probe biding sites of the QRPS and qPCR and ddPCR assays, respectively

A

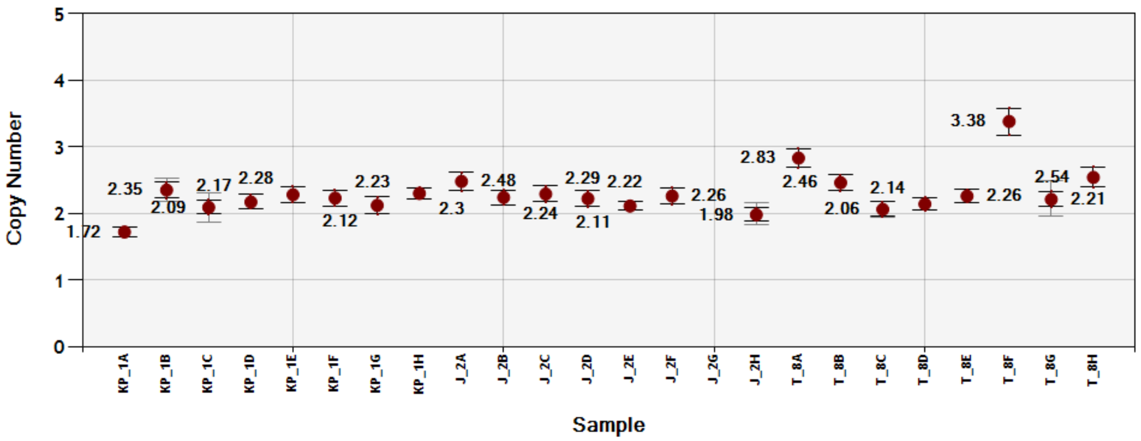

B

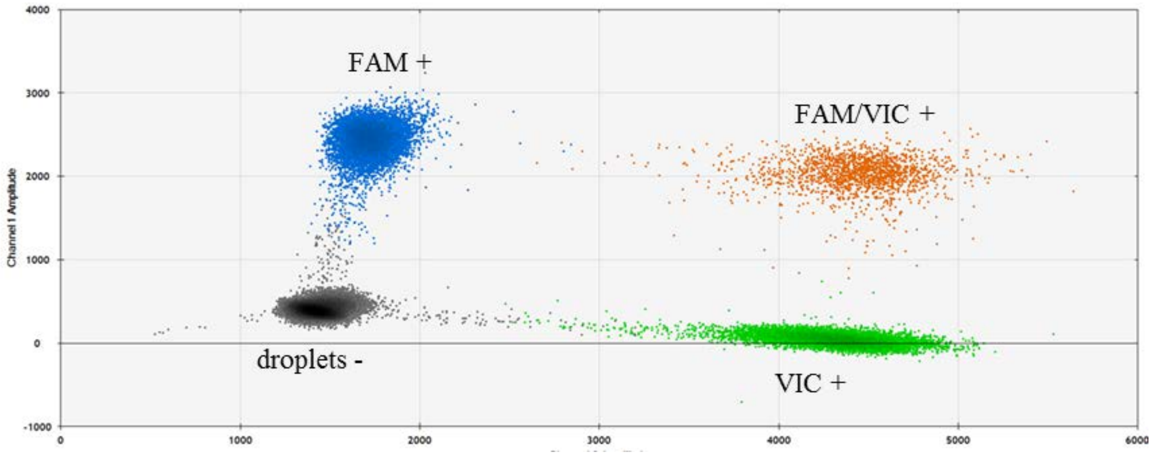

Figure S2. ddPCR CN prediction in Ugandan *Culex quinquefasciatus* mosquitoes A) Merges of replicate calculated CNs. The error bars represent the Poisson 95% confidence interval. B) 2-D amplitude plot of the *Vgsc* and *Pka* showing satisfactory PCR conditions to identify four well defined droplets populations.

A

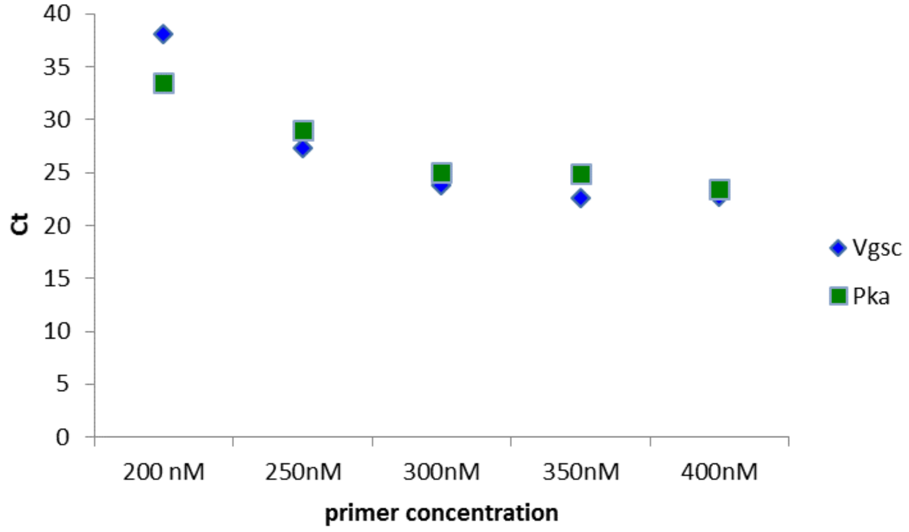

B

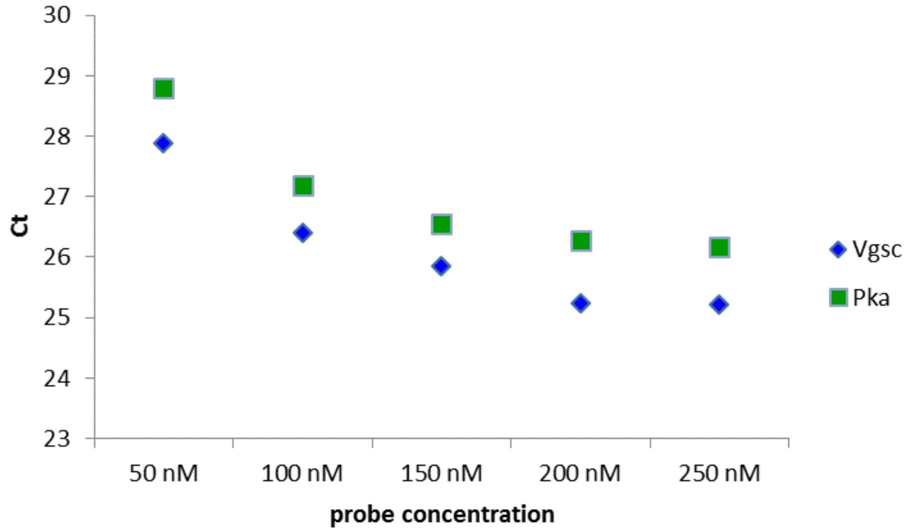

Figure S3. Primer and probe validation. A) Primer set titration to identify concentrations with similar patterns of amplification based on SYBR-GREEN detection. B) Probe titration to identify early and constant Ct values.

A

B

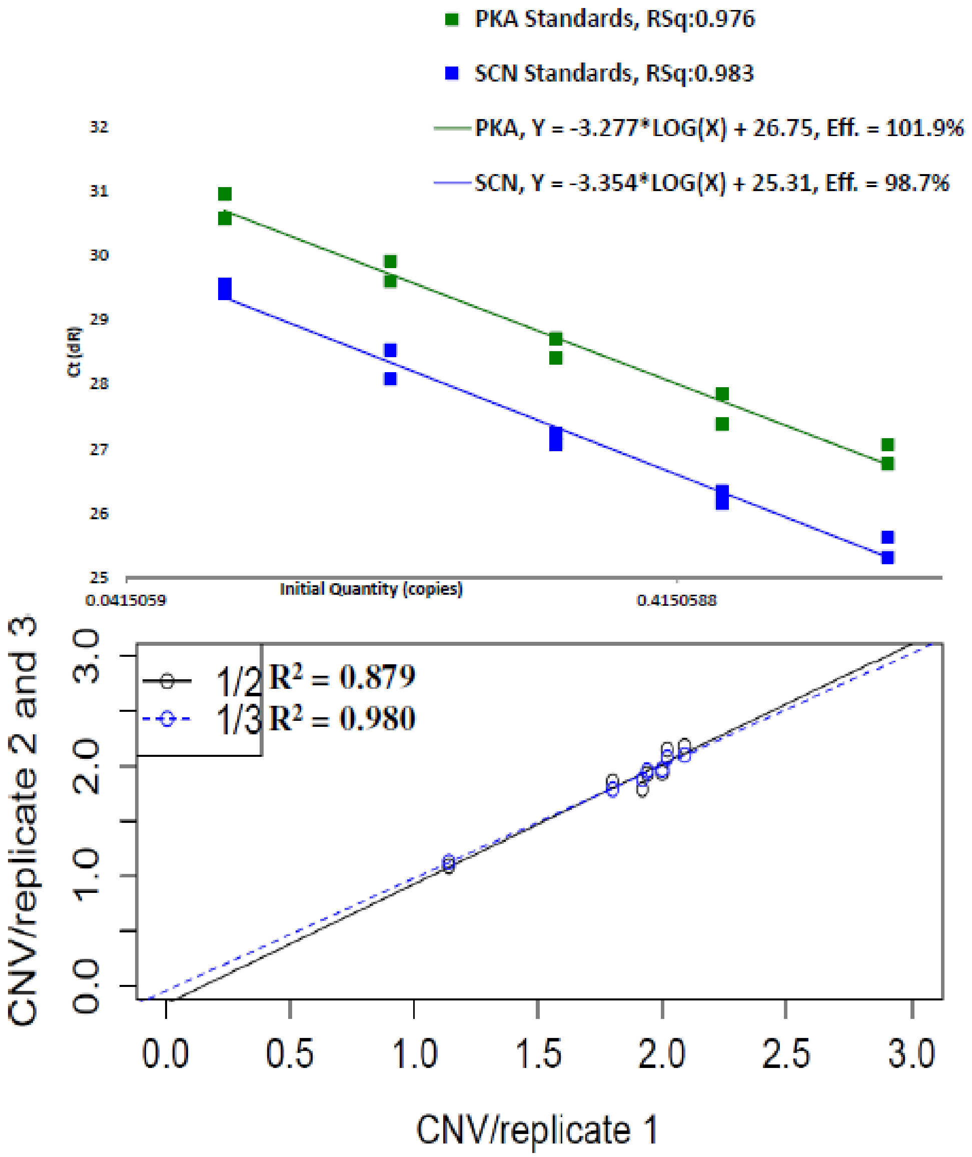

Figure S4. Copy number variation assay validation. A) Efficiency of primers and probes in duplex PCR reactions assessed by standard curve with 2-fold DNA dilution. B) *Vgsc*-CN assay intra-experiment reproducibility comparing triplicates of gDNA with unknown CN in three independent experiments.1/2 and 1/3 correspond to correlation between experiments 1 and 2 and 1 and 3.

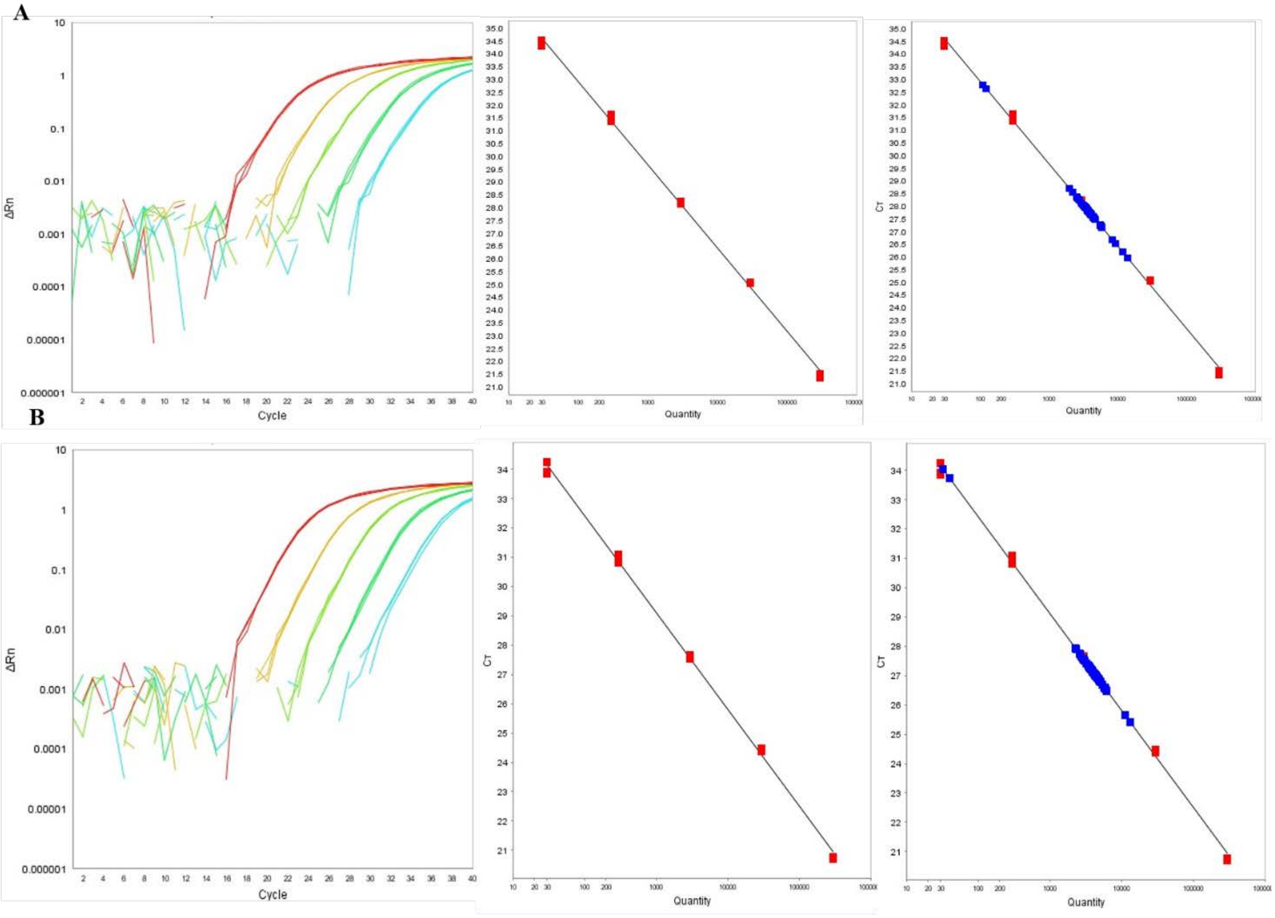

Figure S5. qPCR amplification of the *Vgsc-Pka* plasmid serial dilution for absolute quantification using the qPCR-Std method. Panels A and B show the standard curve amplification for the *Vgsc* and *Pka* genes, respectively. Red squares on the standard curve correspond to the five points of the serial dilution ranging from 3 x 10^5 to 10^1 copies/µl, whereas the blue squares correspond to quantification of samples with unknown copy number.

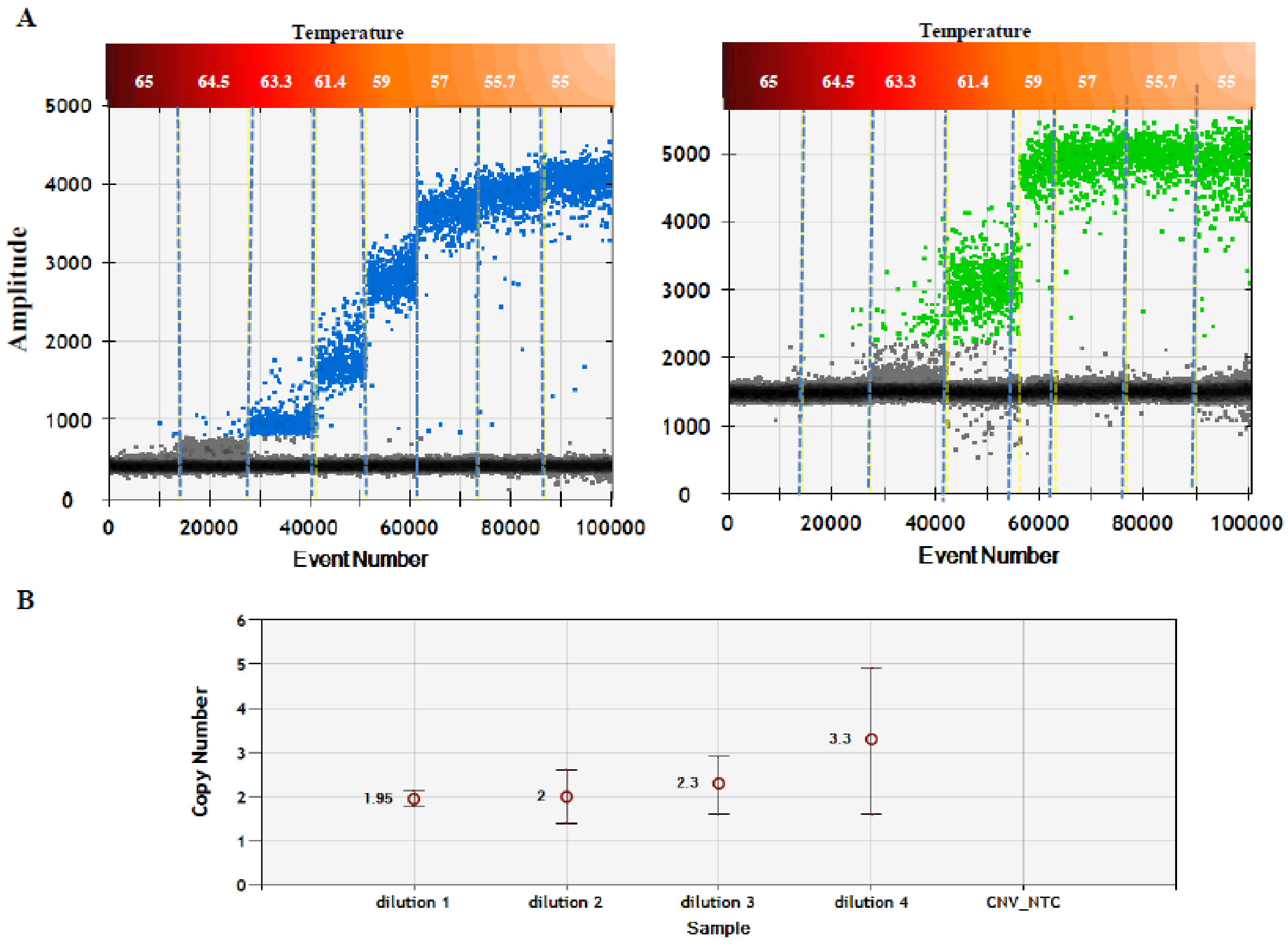

Figure S6. Optimization of annealing temperature and concentration of gDNA for application of the *Vgsc*-CN assay *via* ddPCR analysis. A) Thermal gradient to identify appropriate fluorescent droplet amplitudes for the *Vgsc* (blue dots, FAM channel) and *Pka* (green dots, VIC channel) genes. B) Two-fold *Vgsc-Pka* plasmid dilution to identify DNA concentration for precise CN quantification. Dilutions 1 to 4 correspond to concentrations ranging from 17 x 10^3 copies/µl to 2 x 10^3 copies/µl.

Table S1. Presence of *Vgsc* exons in different *C. quinquefasciatus* (Johannesburg strain) contigs indicates the presence of two *Vgsc* copies.

	COPY 1	COPY 2
Exon 1	AAWU01013212.1	
Exon 2		
Exon 3	AAWU01013208.1	
Exon 4	AAWU01013207.1	
Exon 5	AAWU01013206.1	
Exon 6	AAWU01013204.1	
Exon 7	AAWU01013204.1	
Exon 8	AAWU01013204.1	
Exon 9	AAWU01013204.1	
Exon 10	AAWU01013204.1	
Exon 11	AAWU01013204.1	
Exon 12	AAWU01013202.1	AAWU01037504.1
Exon 13	AAWU01013202.1	AAWU01037504.1
Exon 14		AAWU01037504.1
Exon 15	AAWU01013201.1	AAWU01037503.1
Exon 16	AAWU01013201.1	AAWU01037503.1
Exon 17	AAWU01013201.1	AAWU01037500.1
Exon 18	AAWU01013201.1	AAWU01037500.1
Exon 19 C	AAWU01013201.1	AAWU01037500.1
Exon 19 D	AAWU01013201.1	AAWU01037500.1
Exon 20	AAWU01013201.1	AAWU01037500.1
Exon 21	AAWU01013201.1	AAWU01037500.1
Exon 22	AAWU01013201.1	AAWU01037499.1
Exon 23	AAWU01013201.1	AAWU01037499.1
Exon 24	AAWU01013201.1	AAWU01037499.1
Exon 25	AAWU01013201.1	AAWU01037499.1
Exon 26 K	AAWU01013201.1	AAWU01037498.1
Exon 26 1	AAWU01013201.1	AAWU01037498.1
Exon 27	AAWU01013201.1	AAWU01037496.1
Exon 28	AAWU01013201.1	AAWU01037496.1
Exon 29	AAWU01013201.1	AAWU01037496.1
Exon 30	AAWU01013201.1	AAWU01037496.1
Exon 31	AAWU01013201.1	AAWU01037495.1
Exon 32	AAWU01013201.1	AAWU01037495.1
